# New Insights in Anti-Angiogenesis in Multiple Myeloma

**DOI:** 10.3390/ijms19072031

**Published:** 2018-07-12

**Authors:** Domenico Ribatti, Angelo Vacca

**Affiliations:** 1 Department of Basic Medical Sciences, Neurosciences and Sensory Organs, University of Bari Medical School, Bari 70124, Italy; 2 Department of Biomedical Sciences, and Human Oncology, Section of Internal Medicine and Clinical Oncology, University of Bari Medical School, Bari 70124, Italy

**Keywords:** angiogenesis, anti-angiogenesis, multiple myeloma, tumor growth

## Abstract

Angiogenesis is a constant hallmark of multiple myeloma (MM) progression and involves direct production of angiogenic cytokines by plasma cells and their induction within the bone marrow microenvironment. This article summarizes the more recent literature data concerning the employment of anti-angiogenic therapeutic agents actually used in preclinical models and clinical settings for the treatment of multiple myeloma.

## 1. Angiogenesis in Multiple Myeloma (MM)

Under physiological conditions, angiogenesis depends on the balance of positive and negative angiogenic modulators within the vascular microenvironment. Tumor angiogenesis is linked to switch this balance, and mainly depends on the release by neoplastic cells of growth factors specific for endothelial cells and able to stimulate the growth of the host’s blood vessels [1]. Numerous clinical studies have shown that the degree of angiogenesis or the levels of angiogenic factors are correlated with disease stage, prognosis or response to therapy, suggesting that angiogenesis induction in solid and hematological tumors has a pathophysiologic relevance for disease progression.

Solid tumors progress through an avascular phase (dormant tumor) followed by a vascular phase (angiogenic switch). The role of angiogenesis in the growth of hematological malignancies has become evident since 1994 [2] through the demonstration that tumor progression related to the degree of angiogenesis. Tumor angiogenesis occur through different mechanisms: (i) postnatal vasculogenesis [3]; (ii) vasculogenic mimicry [4]; (iii) vascular cooption [5]; (iv) intussusceptions [6].

Tumor blood vessels, consisting of consist of endothelial cells, mural cells and their enveloping basement membrane, present structural and functional abnormalities. Their leakiness and expression of specific surface molecules facilitates metastatic process.

Angiogenesis is a feature of MM progression through the transition from monoclonal gammopathies of undetermined significance (MGUS) to MM, and has a prognostic potential [7]. It is induced by plasma cells via angiogenic factors released by the cells composing the tumor microenvironment and loss of angiostatic activity in MGUS [8]. It is important to note that already normal plasma cells express a surplus of pro-angiogenic over anti-angiogenic genes which transmits into the ability to induce in vitro angiogenesis. This angiogenic stimulus is further increased in myeloma due to the aberrant expression of pro-angiogenic and down-regulation of anti-angiogenic genes by myeloma cells [9].

Rajkumar et al. [10] confirmed in a cohort of 400 patients that a progressive increase in bone marrow microvascular density occurs across the whole spectrum of plasma cell disorders, including primary amyloidosis, MGUS, smouldering and active MM. Several other reports have described a significant correlation between bone marrow (BM) microvascular density and progression free survival (PFS) and overall survival (OS) in MM patients [11]. Moreover, BM microvascular density decreases significantly in patients achieving a remission, but not in patients who had no response to therapy [12].

Within the bone marrow microenvironment, bone marrow stromal cells (BMSCs), hematopoietic stem cells (HSCs), fibroblasts, osteoblasts/osteoclasts, adipocytes, endothelial precursor cells (EPCs), T lymphocytes, macrophages, and mast cells, increase the concentration of angiogenic factors and matrix degrading enzymes in the bone marrow microenvironment by direct secretion or following stimulation by myeloma cells or by endothelial cells through paracrine interactions [13,14]. BMSCs, osteoclasts, osteoblasts and endothelial cells secrete several factors (Table 1), including vascular endothelial growth factor (VEGF), fibroblast growth factor-2 (FGF-2), tumor necrosis factor alpha (TNF-α), hepatocyte growth factor (HGF), interleukin-6 and -8 (IL-6 and IL-8), osteopontin (OPN), angiopoietin-1 (Ang-1), B-cell activating factor, stromal cell-derived factor 1α (SDF1-α, also known as CXCL12), and various Notch family members, which are further up-regulated by tumor cell adhesion to extracellular matrix proteins and/or BMSCs [13,14]. Macrophages, mast cells and fibroblasts contribute to angiogenesis in MM, in parallel to tumor progression [15,16,17].

In active MM plasma cells secrete VEGF and FGF-2 that induce the cells of the tumor microenvironment to secrete their own VEGF, FGF-2, and HGF, able to recruit and activate the MM-associated macrophages [8]. Moreover, bone marrow macrophages protect MM cells from spontaneous and melphalan-induced apoptosis [19]. In patients with active MM, FACS analysis on isolated bone marrow mononuclear cells revealed higher percentages of CD68+ macrophages than in patients with non-active disease or those with MGUS [10]. Bone marrow macrophages in patients with active MM were similar to paired endothelial cells and contributed to umor angiogenesis through a vasculogenic mimicry [10]. When exposed in vitro to VEGF and FGF-2, tumor macrophages differentiated into cells similar to MM endothelial cells, able to generate in vitro capillary-like networks [10]. Moreover, at confocal laser microscopy tumor macrophages expressed markers of both macrophages and endothelial cells [10].

Bone marrow angiogenesis and mast cell counts are highly correlated in patients with non-active and active MM and in those with MGUS, and both parameters increase simultaneously in active MM [20]. At the ultrastructural level, vessels are lined by mast cells showing numerous and irregularly shaped electron dense granules [9]. Moreover, thick endothelial cells, containing endocytic vesicles, but lacking granules, were connected by a junctional system with the mast cells lining the vessel wall [9]. These ultrastructural findings have been confirmed by confocal laser microscopy using double anti-tryptase (to mark mast cells) and anti-FVIII-RA (to mark endothelial cells) antibodies. In MM, vessels displayed characteristics of both mast cells and endothelial cells, while in MGUS, the vessels were uniformly stained with the endothelial marker [9]. Overall, these data suggest that mast cells also contribute to MM neovascularization.

Tumor-associated fibroblasts (TAFs) were increased in patients with active MM compared to those in remission and those with MGUS. The cells displayed an activated phenotype, and produced high levels of TGF-β, IL-6, SDF-1α, and IGF-1 [15]. Moreover, TAFs showed a heterogeneous phenotype which entailed their origin from resident fibroblasts, and from endothelial cells and HSCs via endothelial-mesenchymal transition, and from mesenchymal stem cells (MSCs) via mesenchymal transition, all induced by both tumor-associated fibroblasts and MM plasma cells. Active MM fibroblasts induces chemotaxis, adhesion, proliferation and apoptosis-resistance of MM cells through the release of cytokines and cell-to-cell contact that was inhibited by blocking CXCR4, β3 and β7 integrins, and fibronectin. Experiments performed in syngeneic 5T33MM and xenografted mouse models showed that MM cells induced the tumor fibroblasts recruitment and expansion which, in turn, favored tumor initiation and progression as well as angiogenesis [15].

Circulating endothelial cells and endothelial precursor cells (EPCs) contribute to the neovascularization, and the presence of EPCs suggests that vasculogenesis may also contribute to the full MM vascular tree [14]. We have demonstrated that in patients with active MM, plasma cells and stromal cells in the bone marrow microenvironment recruit HSCs, and induce their transformation into mature MM endothelial cells [21]. In fact, when HSCs of MM patients were incubated with VEGF, FGF-2 and insulin-like growth factor (IGF), cells differentiate into endothelial cell-like cells expressing typical endothelial markers, such as factor VIII-related antigen (FVIII-RA), VEGFR-2 and VE-cadherin, and form capillary-like networks in vitro [21].

The deregulated interactions between MM cells and other cells of the microenvironment are at the basis of the clinical manifestations of the disease, including osteolytic bone lesions, hypercalcemia, and suppressed haematopoietic functions. The vascular niche is comprised of vasculature forming a conduit which enables MM cells both to leave the osteoblastic niche and enter the vascular system via transendothelial migration, hence to return to the bone marrow via homing mechanisms. In this context, endothelial cells, pericytes, and smooth muscle cells create a microenvironment that recruits EPCs, MSCs and HSCs. The vascular niche is a site required for the differentiation and maturation of HSCs via both the production and secretion of various cytokines and growth factors, as well as via direct cell-cell contact. HSCs in turn prolong survival of bone marrow endothelial cells by secreting endothelial cell growth factors [22].

A comparative gene expression profiling of endothelial cells derived from patients with MM and with MGUS has been carried out [23]. Twenty-two genes were found differentially expressed (14 down-regulated and 8 up-regulated) at relatively high stringency in MM endothelial cells. Deregulated genes were mostly involved in extracellular matrix formation and bone remodeling, cell adhesion, chemotaxis/spread, angiogenesis, resistance to apoptosis, and cell-cycle regulation. Validation was focused on *DIRAS3*, *SERPINF1*, *SRPX*, *BNIP3*, *IER3*, and *SEPW1* genes, which were not previously found to be functionally correlated to the angiogenic phenotype of MM endothelial cells. The siRNA for three up-regulated genes (*BNIP3*, *IER3*, and *SEPW1*) affected endothelial cell proliferation, apoptosis, adhesion/spread, and capillary tube formation. Two apoptosis-related genes were up-regulated in MM endothelial cells: BNIP3 belongs to the Bcl-2 family, and IER3 is a member of the “immediate early response gene” family induced by the anti-apoptotic factor nuclear factor κB in response to the tumor necrosis factor-α and ligand-mediated FAS. Coordinated anti-apoptotic mechanisms are activated in MM endothelial cells and contribute to their over-angiogenic phenotype.

Recombinant human erythropoietin (rHuEpo) is involved in the regulation of the angiogenic response in MM through a direct effect on macrophages and endothelial cells isolated from the bone marrow of patients with MM [24,25]. Interactions between plasma cells and BMSCs are modulated by specific cytokines, receptors and adhesion molecules, responsible for the proliferation, migration and survival of plasma cells, disease progression and acquisition of drug resistance [13]. Although it has well established that myeloma cells drive angiogenesis by the secretion of angiogenic factors, there is also evidence for a loss of anti-angiogenic activity on the part of bone marrow plasma cells with disease progression [12,26].

## 2. Anti-Angiogenesis in Multiple Myeloma

A number of antiangiogenic therapeutic strategies have been evaluated in myeloma with varying degrees of efficacy but the results overall further confirm the role of angiogenesisin the pathogenesis of myeloma. In MM treatment, thalidomide, lenalidomide and bortezomib have changed clinical practice for both the newly presented and the relapsed patients.

### 2.1. Thalidomide

The anti-angiogenic properties of thalidomide led to the consideration of its use in MM [27]. In addition to its anti-angiogenic activity due to its inhibition of secretion of VEGF and IL-6 [28], thalidomide enhances T cell- and NK cell-mediated immunological responses, induces caspase-8 mediated apoptosis, and down-regulates IL-6 production within the BM microenvironment [29,30]. Based on the increasing interest for the use of thalidomide as an anti-angiogenic agent, Barlogie and colleagues initiated a compassionate-use trial of “anti-angiogenic therapy.” Barlogie conducted a trial including 84 patients and had 32% of patients respond, making it the first new drug with single-agent activity for myeloma in more than three decades [31].

### 2.2. Immunomodulatory Drugs

Subsequently to thalidomide, a series of immounomodulatory drugs (IMiDs) have been developed. Pomalidomide is the most potent IMiD, having 100 times the strength of thalidomide and 10 times that of lenalidomide [32].

Lenalidomide inhibits VEGF-induced PI3K-Akt pathway signaling and HIF-1α expression [32], exerts an anti-tumor necrosis factor alpha (TNFα) activity, modulates the immune response stimulating T cells and NK cells activities, induces apoptosis of tumor cells, and decreases the binding of MM cells to BMSCs [30,33,34,35,36]. Moreover, lenalidomide alter the balance of bone resorption by inhibiting osteoclast formation [37,38]. Lenalidomide inhibits MM plasma cells-induced angiogenesis in vivo in the chorioallantoic membrane (CAM) assay and endothelial cells-induced angiogenesis in vitro in the Matrigel assay, inhibits MM endothelial cell migration, and down-regulates key genes and proteins related to MM angiogenesis [39]. In clinical trials with lenalidomide, the absence of a significant reduction in bone marrow neovascularization after treatment suggests that the anti-angiogenic effect may not play a major role in this setting [40].

Lenalidomide can be taken orally, produced only modest side effects, and was an important addition to the treatment for multiple myeloma. Lenalidomide was approved in the USA in 2006 for the treatment of relapse refractory multiple myeloma (RRMM) [41]. When it was combined with dexamethasone 90% of newly diagnosed patients responded [42]. A retrospective analysis of clinical trials demonstrated an higher overall response rate (ORR), longer time to progression (TTP), and progression-free survival (PFS) for patients treated with lenalidomide and dexamethasone, compared to the those treated only with dexamethasone [43]. Lenalidomide sensitizes MM plasma cells to bortezomib [44], and lenalidomide/bortezomib/dexamethasone produced responses in 84% of relapsed/refractory patients, including complete response or near complete response in 21% [45], and produced responses in 98–100% of newly diagnosed MM patients [46]. Lenalidomide is partly eliminated in urine and it is therefore necessary to adjust the dose depending on renal function.

Pomalinomide is active against MM cell lines in case of bortezomib and lenalidomide resistance and inhibits angiogenesis by targeting VEGF and HIF-1α [47]. Pomalidomide is effective in RRMM patients both as a single agent [46,48,49] and in combination with low-dose dexamethasone, even in patients refractory to other IMiDs and/or bortezomib [41,50,51,52]. Comparison of pomalidomide plus low-dexamethasone to high-dose dexamethasone demonstrated a longer PFS and a better response rate in the pomalidomide group [53]. The pharmacokinetics of pomalidomide does not seem to be affected by renal impairment.

### 2.3. Bortezomib and Second-Generation Proteasome Inhibitors

Bortezomib is currently used in the treatment of myeloma and in addition to its proteosomal inhibitory effects it exerts inhibitory effects on endothelial cell proliferation and migration as well as to downregulate VEGF and Ang expression by endothelial cells. Bortezomib induces endothelial cell apoptosis [54], inhibits VEGF, IL-6, Ang-1 and Ang-2 and IGF-1 secretion in BMSCs and endothelial cells derived from MM patients [55,56]. Bortezomib is approved for the treatment of MM in the relapsed setting post-transplant or as a second line treatment in patients unsuitable for transplantation. Due to its novel mechanism of action, bortezomib has been shown to induce responses in previously refractory patients (including those with poor risk cytogenetics), and results in an increased progression free and overall survival in relapsed patients when compared with dexamethasone treatment alone. It is well tolerated and peripheral neuropathy is the most common dose limiting toxicity and thrombocytopenia can generally be managed with platelet transfusions. Bortezomib shows a synergistic effect in combination with dexamethasone and also sensitizes myeloma cells to the effects of other chemotherapeutic agents [57]. Carfilzomib is a second-generation proteasome inhibitor with a similar anti-angiogenic potential like bortezomib. Carfilzomib binds selectively with the chymotrypsin-like site of the proteolytic core and is currently examined in different doses and regimens in RRMM as in newly diagnosed MM [58]. Inducing irreversible proteasome inhibition, carfilzomib not only demonstrates greater preclinical antitumor activity [59], but it is also effective in cell lines already resistant to bortezomib [59]. Ixazomib is another reversible second-generation proteasome inhibitor that is active in bortezomib-resistant myeloma cells, despite structural similarities [60]. It has become the first orally bioavailable proteasome inhibitor to be approved for the treatment of recurrent multiple myeloma in the USA [59].

### 2.4. Bishosphonates

The administration of bisphosphonates, inhibitors of osteoclasts activity, exerts an antiangiogenic activity. Therapeutic doses of zoledronic acid markedly inhibit in vitro proliferation, chemotaxis and angiogenesis of MM endothelial cells and in vivo angiogenesis in the CAM assay [61]. Bortezomib and zoledronic acid display distinct and synergistic activities on bone marrow macrophages in MM patients, inhibiting macrophage proliferation, adhesion, migration, and expression of angiogenic cytokines, angiogenesis on Matrigel, VEGFR-2 and ERK1/2 phospho-activation as well as nuclear factor kB (NF-kB) [62]. These drugs synergistically inhibit macrophage vasculogenesis on the Matrigel surface and the expression of FVIII-RA, Tie2/Tek, and VEGFR-2/VE-cadherin, expression of cell transdifferentiation into endothelial-like cells. Both drugs reduce phospho-activation of VEGFR-2 and ERK1/2 and NFKB activity [62]. These data provide evidence that the exposure of bone marrow macrophages during the treatment with bortezomib and zoledronic acid impacts their angiogenic and vasculogenic properties, suggesting that these cells may be considered as a target of both drugs in MM patients, demonstrating that when macrophages pre-treated with bortezomib were co-cultured with MM cell lines, the proliferative index of these latter decreased significantly [63].

## 3. New Insights 

In a xenograft MM mouse model, the administration of Pazopanib, an orally available inhibitor of VEGFR-1, VEGFR-2, and VEGFR-3 led to higher survival, reduced tumor growth, and angiogenesis. However, the tumors regrew after the treatment ceased [64]. Futhermore, Pazopanib was ineffective in a phase II clinical trial with patients with relapsed and refractory MM [65].

IL-12 receptor B2 (IL-12Rβ2) is expressed in primary MM cells but down-regulated compared with normal polyclonal plasmablastic cells and plasma cells [66]. Moreover, IL-12 reduced the pro-angiogenic activity of primary MM cells in vitro and decreased significantly the tumorigenicity of NCI-H929 cell line in SCID/NOD mice by inhibiting cell proliferation and angiogenesis [66].

Coluccia et al. [67] demonstrated that platelet derived growth factor (PDGF)-BB/PDGF receptor beta (PDGFRβ) promoted the transcription of MMEC-proangiogenic factors, such as VEGF, FGF-2, and IL-8. Moreover, a prolonged exposure of MM endothelial cells to dasatinib, an oral bioactive PDGFRβ/Src tyrosine kinase inhibitor, annulled their ability to respond to VEGF, preventing the expression of endogenous VEGF in a time-dependent manner, and the levels of secreted VEGF in the conditioned medium of MM endothelial cells in a dose-dependent manner.

Roccaro et al. [68] demonstrated that microRNA-15a/-16, which are downregulated in MM plasma cells as compared to normal cells, exert an antiangiogenic activity, reducing VEGF secretion from MM cells at the protein level, thereby reducing MM plasma cell proangiogenic activity on endothelial cells and these data were further confirmed in vivo in the CAM assay. Therefore, the antiangiogenic role of microRNA-15a and –16 may contribute, at least in part, to their anti-MM activityTargeting the SDF1-α/CXCR4 pathway with CXCR4 antagonist AMD3100 can mobilize MM cells in the circulation and render them sensitive to anti-myeloma treatment [69]. The combination of ADM3100 with bortezomib led to a decrease in tumor burden in vivo [69]. Ulocuplumab, a monoclonal anti-CXCL4 antibody, as well as neutralization of SDF1 by Olaptesed-pegol (PEGylated mirror-image l-oligonucleotide), inhibit myeloma cell dissemination and suppress the CXCR4-driven EMT-like [70].

Sorafenib, a multi-targeted receptor tyrosine kinase inhibitor, targeting VEGFR-2, VEGFR-3, RAF, PDGFR-B, Flt-3, and cKit exerts a significant anti-MM activity and synergize with other anti-MM drugs [71]. Sorafenib has also been tested in patients with refractory or recurrent MM and was effective in two patients who achieved a partial response and a continuous stable disease [72]. Natalizumab, a selective adhesion molecule inhibitor, which binds α4 integrins inhibits VEGF secretion and angiogenesis and enhances the anti-MM activity of bortezomib and dexamethasone [73]. Akt pathway is crucial for MM survival and drug resistance and has been proposed as a target for future molecular therapies [74].

HGF/cMET enhances the expression of VEGF/VEGFR-2 in MM endothelial cells [75,76] . In vitro and in vivo studies of a selective cMET inhibitor, SU 11274, alone or in combination with bortezomib, lenalidomide and dexamethasone, indicate that the HGF/cMET pathway is a new therapeutic target for RRMM [75,76]. Umezu et al. [77] found that exosomal miR-340 derived from BMSCs inhibited myeloma related angiogenesis via the HGF/cMET signaling pathway in endothelial cells. The use of anti-murine VEGFR-2 antibody DC101 prevented the mobilization of EPCs and delayed tumor progression in the early stages of MM, but was ineffective when used during MM [78]. Clinical trials using DARPin MPO250 in RRMM are already running (e.g., NCT03136653) and preliminary data in patients with RRMM have been presented at the 2018 European Hematology Association (EHA)meeting in Stockholm (available online: http:// ehaweb.org).

Rao et al. [79] investigated the anti-angiogenic effect of MPO250, a multi-domain designed ankyrin repeat protein (DARPin) drug which binds simultaneously VEGF and HGF, in MM. They demonstrated that MPO250 reduces VEGFR-2 and cMET phosphorylation and affects their downstream signaling cascades. Moreover, MPO250 influences the secretory profile of MM endothelial cell, and inhibits in vitro and in vivo angiogenic activities of MM endothelial cells at higher extent than anti-VEGF or anti-HGF neutralizing monoclonal antibodies. Finally, in the syngeneic 5T33MM tumor model, MPO250 decreases the microvessel density and in combination with bortezomib, lowers the percentage of idiotype-positive cells and the serum levels of M protein.

Lamannuzzi et al. [80] evaluated mammalian target of rapamycin (mTOR) activation in endothelial cells from patients with MM and with MGUS. They demonstrated an increase of mTOR phosphorylation at the Ser2448 associated to a higher expression of RICTOR and to a rise in AKT phosphorylation at Ser473 in MM endothelial cells, indication that mTOR complex 2 (mTORC2) is more activated than mTORC1. On the contrary, MGUS endothelial cells showed an increase of SGK1 phosphorylation, expression of mTORC1 activation. These data were supported by the higher activation of mTORC2 downstream effectors, suggesting a major role of mTORC2 in the angiogenic switch to MM. Moreover, specific inhibition of mTOR activity through siRNA targeting RICTOR and dual mTOR inhibitor PP242 reduced the MM endothelial cells angiogenic functions, including cell migration, chemotaxis, adhesion, invasion, in vitro morphogenesis on Matrigel, and cytoskeleton reorganization. Finally, PP242 showed anti-angiogenic effects in vivo in the CAM and Matrigel plug assays, and exhibited a synergistic effect with lenalinomide and bortezomib.

## 4. Concluding Remarks

MM is the second most common blood-based malignancy, affecting approximately 20,000 new patients each year. More effective treatment options for MM are urgently needed. Disease relapse is inevitable and MM remains incurable. Anti-cancer drugs including novel therapies in monotherapy have debilitating side effects, and these treatments only benefit a minority of patients. The identification of subgroups of patients who will derive the most benefit from a drug with manageable drug-related toxicity is an important step towards improved response rates, safety and survival.

In addition to using anti-angiogenic agents that target more than one pro-angiogenic factor, another strategy is to use combined modalities. The abnormalities of the tumor vasculature and the impaired blood flow they cause result in an abnormal microenvironment that is characterized by hypertension, hypoxia and acidosis. These characteristics pose a significant barrier to cancer therapy, with leaky vessels impairing the delivery of therapeutics to the tumor and hypoxia rendering cells resistant to both radiation and many cytotoxic drugs. Therefore, an approach to normalize tumor vessels may correct the tumor microenvironment, making it more susceptible to therapy. Anti-angiogenic therapies have been shown to normalize tumor vasculature and can therefore improve treatment efficacy when co-administered with other therapies [81].

The median survival for patients with MM has almost doubled since the introduction of thalidomide, lenalidomide and bortezomib, along with autologous stem cell transplant [82]. Various combinations of thalidomide, lenalidomide and bortezomib, when combined with melphalan and prednisone or other drugs as first line therapies or in relapsed treatments have given higher overall response rates (ORRSs) and superior OS. However, most MM patients will still relapse and require a change in therapy [83]. In this context, treatment option is represented by monoclonal antibodies targeting CD38 (including Daratumab, Isatuximab, MOR202) and SLAMF7 [84,85]. In 2015, the anti-CD38 monoclonal antibody Daratumab has been approved by the US Food and Drug Administration (FDA), and a second anti-CD38 monoclonal antibody, Isatuximab, shows single-agent activity in patients with relapsed and refractory MM [86]. Clinical synergy with immunomodulatory agents has also been reported using SLAMF7 monoclonal antibody, Elotuzumab [87].

Resistance to anti-angiogenic agents is a clinically significant problem. Different approaches to circumvent this problem have been established, including the use of multiple-targeted anti-angiogenic agents and/or combined-modality treatment strategies. It would appear that the future of angiogenic inhibitors lies in the intelligent combination of multiple targeted agents with other angiogenic inhibitors to maximize therapeutic effect.

In conclusion, despite impressive performances in animal models, anti-angiogenic drugs are not performing nearly as well in humans. Anti-angiogenic treatments lead to a limited increase in PFS, followed by a relapse in tumor angiogenesis and growth. Further studies to optimize treatment regimens and to increase our understanding of tumor angiogenesis and the mechanisms underlying the development of resistance are required. The most important objective is to establish validated biomarkers with the aim to personalize treatments and select responding patient sub-populations.

## Figures and Tables

**Table 1:** Main angiogenic factors in active multiple myeloma (MM) [18].

Angiogenic Factor	Origin	Receptors	Experimental Observation
VEGF (Vascular endothelial growth factor)	Multiple myeloma (MM) Plasma cells (MMPCs) Multiple myeloma ECs (MMECs)	1. VEGFR-2 on endothelial cells (ECs) 2. VEGFR-1 on bone marrow stromal cells (BMSCs) 3. VEGFR-3 on MMPCs	Promotes MMECs and BMSCs proliferation, growth and chemotaxis Promotes IL-6 and VEGF secretion by BMSCs Up-regulated in active MM Its expression correlates with bone marrow microvascular density
FGF-2 (Fibroblast growth factor-2)	MMPCs	FGF-2R-2	Promotes MMECs and BMSCs proliferation, growth and chemotaxis Promotes IL-6 and VEGF secretion by BMSCs Up-regulated in active MMIts expression correlates with bone marrow microvascular density
HGF (Hepatocyte growth factor)	MMPCs	c-Met	Identified in MM cell lines Up-regulated in active MM
Ang-1 (Angiopoietin-1)	MMPCs	Tie-2/Tek	Up-regulated in active MM Its expression correlates with bone marrow microvascular density Stabilizes nascent vessels by tightening endo-periendothelial cell interactions
Ang-2 (Angiopoietin-2)	MMPCs	Tie-2/Tek	Angiogenic in the presence of VEGF via loosening of periendothelial cells
IGF-1 (Insulin-like growth factor-1)	MMPCs (BMSCs) MMECs	IGF-1R	Stimulates MMPCs to secrete VEGF Up-regulated in active MM Promotes MMECs and BMSCs proliferation, growth and chemotaxis
IL-8 (Interleukin-8)	BMSCs MMECs	IL-8R	Up-regulated in active MM
OPN (Osteopontin)	MMPCs BMSCs MMECs	CD44	Its expression correlates with bone marrow microvascular density
MMP-2/9 (Matrix metalloproteinases-2/9)	MMPCs BMSCs MMECs	Collagen isoforms	Up-regulated in active MM MMP-2 expression is stronger
PDGF-BB (Platelet-derived growth factor-BB)	MMPCs	PDGFRβ	This pathway was selectively induced by VEGF Recruits smooth muscle cells around nascent endothelial channels
ADM (Adrenomedullin)	MMPCs	ADMR	Induces in vitro angiogenesis
TNF-α (Tumor necrosis factor-α)	MMPCs BMSCs MMECs	TNF-αR	Stimulation of IL-6 secretion, bone resorption and expression of adhesion molecules
TGF-β1 (Transforming growth factor-β1)	MMPCs BMSCs MMECs	TGF-β1R	Stabilizes nascent vessels by stimulating ECM production
CXCL8/IL8 (CXC-chemokine CXCl8/IL8)	MMECs	CXCR2	Takes part in a paracrine loop between MMECs and MMPCs to mediate MMPCs proliferation and chemotaxis
CXCL11/I-TAC (CXCL11-interferon-inducible T-cell α chemoattractant)	MMECs	CXCR3	Takes part in a paracrine loop between MMECs and MMPCs to mediate MMPCs proliferation and chemotaxis
CXCL12/SDF-1α (CXCL12/stromal cell-derived factor-1α)	MMECs	CXCR4	Takes part in a paracrine loop between MMECs and MMPCs to mediate MMPCs proliferation and chemotaxis

## Abbreviations

MM	multiple myeloma
BM	bone marrow
PFS	progression free survival
OS	overall survival
HSC	hematopoietic stem cell
BMSCs	bone marrow stromal cells
EPCs	endothelial precursor cells
VEGF/R	vascular endothelial growth factor/receptor
FGF-2/R	fibroblast growth factor-2/ receptor
TNF-α	tumor necrosis factor alpha
HGF	hepatocyte growth factor
IL-6 and IL-8	interleukin-6 and -8
OPN	osteopontin
Ang-1	angiopoietin-1
Tie-2/Tek	angiopoietin-1 receptor
SDF1-α or CXCL12	stromal cell-derived factor 1α
MMPCs	multiple myeloma plasma cells
MMECs	multiple myeloma ECs
HGF	hepatocyte growth factor
c-Met	hepatocyte growth factor receptor
Ang 1	angiopoietin-1
Ang-2	angiopoietin-2
IGF-1	insulin-like growth factor-1
OPN	osteopontin
MMP-2/9	matrix metalloproteinases-2/9
PDGF-BB	platelet-derived growth factor-BB
ADM	adrenomedullin
TGF-1	transforming growth factor-1
CXCL11/I-TAC	CXCL11-interferon-inducible T-cell chemoattractant
MSCs	mesenchymal stem cells
BNIP3	BCL2 Interacting Protein 3
*Bcl-2*	*B-cell lymphoma 2*
IER3	immediate early response 3
FAS	fas cell surface death receptor
rHuEpo	recombinant human erythropoietin
IMiDs	immounomodulatory drugs
RRMM	refractory multiple myeloma
ORR	higher overall response rate
TTP	longer time to progression
PFS	progression-free survival
FVIIIRA	factor VIII-related antigen
ERK	Extracellular signal–regulated kinases
NFKB	nuclear factor kappa-light-chain-enhancer of activated B cells
PDGF	platelet derived growth factor
PDGFRβ	BB/PDGF receptor beta
AMD3100	CXCR4 Antagonist
SU11274	cMET inhibitor
DC101	anti-murine VEGFR-2 antibody
DARPin	multi-domain designed ankyrin repeat protein
mTOR	mammalian target of rapamycin
mTORC	mTOR complex
CAM	chorioallantoic membrane
